# Impairments in brain perfusion, executive control network, topological characteristics, and neurocognition in adult patients with asymptomatic Moyamoya disease

**DOI:** 10.1186/s12868-021-00638-z

**Published:** 2021-05-12

**Authors:** Shihao He, Ziqi Liu, Yanchang Wei, Ran Duan, Zongsheng Xu, Cai Zhang, Li Yuan, Tian Li, Ning Ma, Xin Lou, Xiaoyuan Liu, Rong Wang

**Affiliations:** 1 Department of Neurosurgery, Beijing Tiantan Hospital, Capital Medical University, No. 119 South 4th Ring West Road, Fengtai District, Beijing, 100070 People’s Republic of China; 2 Center of Stroke, Beijing Institute for Brain Disorders, Beijing, 100069 China; 3 Department of Neurosurgery, Peking University International Hospital, Beijing, 102206 China; 4 State Key Laboratory of Cognitive Neuroscience and Learning & IDG/Mc Govern Institute for Brain Research, Beijing Normal University, Beijing, 100875 China; 5 Department of Radiology, Chinese PLA General Hospital, Beijing, 100853 China

**Keywords:** Moyamoya disease, Brain perfusion, Neurocognition, Asymptomatic, Cerebral blood flow, Functional magnetic resonance imaging, Brain network

## Abstract

**Background:**

Asymptomatic Moyamoya disease (MMD) impairs hemodynamic and cognitive function. The relationship between these changes, cerebral blood flow (CBF), and network connectivity remains largely unknown. The aim of this study was to increase understanding of the relationship between CBF, functional networks, and neurocognition in adults with asymptomatic MMD. We compared CBF and functional status in 26 patients with MMD and 20 healthy controls using arterial spin labeling and resting state functional magnetic resonance imaging sequences. At the same time, a detailed cognitive test was performed in 15 patients with no cerebral or lumen infarction who were selected by magnetic resonance imaging-T2 FLAIR screening.

**Results:**

Compared to the controls, the patients showed varying degrees of decline in their computational ability (simple subtraction, *p* = 0.009; complex subtraction, *p* = 0.006) and short-term memory (*p* = 0.042). The asymptomatic MMD group also showed decreased CBF in the left anterior central and left inferior frontal gyri of the island flap with multiple node abnormalities in the brain network and reduced network connectivity. There was a significant association of these changes with cognitive decline in the MMD group.

**Conclusions:**

In patients with asymptomatic MMD, disturbance of CBF and impaired brain network connections may be important causes of cognitive decline and appear before clinical symptoms.

*Clinical trial registration-URL*: http://www.chictr.org.cn

**Unique identifier**: ChiCTR1900023610

**Supplementary Information:**

The online version contains supplementary material available at 10.1186/s12868-021-00638-z.

## Background

Moyamoya disease (MMD) is a rare cerebrovascular disease of unknown etiology characterized by chronic progressive distal internal carotid artery stenosis or occlusion [1, 2]. In addition to the clinical finding that patients with MMD are prone to cerebrovascular accidents, they may also experience loss of higher cognitive function [3–5]. Many studies have shown that stroke is an important cause of cognitive impairment in these patients [6, 7]. However, our previous study found that cognitive impairment occurred not only in patients with MMD and a history of cerebral infarction or other cerebrovascular events but also in patients with asymptomatic MMD [8].

Studies have shown that only 1.5–17.8% of patients with MMD are asymptomatic [9]. However, with the recent emphasis on physical examinations and the development of non-invasive diagnostic methods such as magnetic resonance imaging (MRI) and magnetic resonance angiography, it is now believed that the incidence of asymptomatic MMD may be higher than previously thought [10]. Evaluation of cognitive function in asymptomatic patients without cerebrovascular events may be important in revealing the cognitive effects of the disease itself.

ASL is a new cerebral hemodynamic evaluation technique that has been clinically adopted in recent years. Its advantage lies in the absence of radiation and exogenous contrast agents. In addition, there have been a report showing that although the ASL value was lower than the resting ^123^I-N-isopropyl-p-iodoamphetamine value, there was a significant relationship between these two values, suggesting that ASL-MRI perfusion imaging can be used to identify cerebral perfusion [11]. Functional neuroimaging can reveal brain activity and is important in the study of neurological diseases [12]. Changes in functional connectivity in several neurological and psychiatric disorders have provided pathological perspectives on the global and local indices of brain networks that can be used to explain some of the cognitive deficits associated with these diseases [13–15]. However, few studies have investigated whether these changes in brain networks are associated with changes in cognitive function in patients with MMD, especially if asymptomatic.

This study evaluated the functional performance of the brain in patients with MMD by analyzing brain perfusion, independent network components, neurocognition, and topology using functional MRI (fMRI), arterial spin labeling (ASL), and resting state sequences.

## Results

### Patient characteristics and neuropsychological evaluation

We enrolled 26 patients with asymptomatic MMD and 20 healthy controls. In total, 15 patients underwent T2 imaging and complete cognitive testing. There was no significant difference in years of education, sex distribution, or age between the 15 patients and the controls (Table 1). Compared with the control group, the asymptomatic MMD group showed a decrease in mental performance; however, the difference was not statistically significant (SPM, *p* = 0.06). There was a significant decrease in patients' mathematical ability (SUB, *p* = 0.009; COMSUB, *p* = 0.006). There was also a significant decrease in short-term memory for words in the asymptomatic MMD group compared with the control group (WORDM, *p* = 0.042).

**Table 1:** Demographic characteristics and cognitive test scores in the two study groups

	MMD group (*n* = 15)	Control group (*n* = 20)	*p* value	Effect size	Cohen’s d
Sex (M:F)	10:05	13:07	0.918		
Age, years	41.27 ± 10.36	48.95 ± 12.78	0.066	− 0.31	− 0.66
Education, years	10.53 ± 2.26	11.10 ± 3.82	0.588	− 0.09	− 0.18
Neurocognition score					
SPM	15.93 ± 6.94	21.45 ± 9.13	0.06	− 0.32	− 0.68
ROT	14.13 ± 7.28	18.15 ± 12.67	0.246	− 0.19	− 0.38
VWM1	6.93 ± 1.58	7.60 ± 1.73	0.25	− 0.20	− 0.40
VWM2	5.00 ± 1.56	6.20 ± 2.22	0.083	− 0.29	− 0.62
SUB	30.73 ± 11.52	40.70 ± 9.57	0.009	− 0.43	− 0.94
COMSUB	12.00 ± 6.09	19.60 ± 9.32	0.006	− 0.43	− 0.96
WORDM	54.87 ± 14.48	64.40 ± 12.22	0.042	− 0.34	− 0.71
PICTM	72.47 ± 6.97	73.90 ± 5.64	0.506	− 0.11	− 0.23
Medical history, n (%)					
Hypertension	4 (26.7)	5 (25)	0.911		
Diabetes mellitus	2 (13.3)	2 (10)	0.759		
Smoking history	3 (20)	3 (15)	0.698		
Alcohol consumer	3 (20)	2 (10)	0.403		
Suzuki stage					
Left					
1	3 (20)				
2	5 (33.3)				
3	5 (33.3)				
4	1 (6.7)				
5	1 (6.7)				
6	0				
Right					
1	3 (20)				
2	4 (26.7)				
3	5 (33.3)				
4	2 (13.3)				
5	1 (6.7)				
6	0				

The data are shown as the number (percentage) unless otherwise indicated. Mean values are presented with the standard deviation

*F* female, *M* male, *MMD* asymptomatic Moyamoya disease, *SPM* Raven’s Standard Progressive Matrices, *ROT* Mental rotation, *VWM1* Verbal working memory-forward digit span task, VWM2 Verbal working memory-backward digit span task, *SUB* simple subtraction, *COMSUB* complex subtraction, *WORDM* word-memory, *PICTM* picture-memory

### Difference in CBF between the study groups

**Fig. 1:**
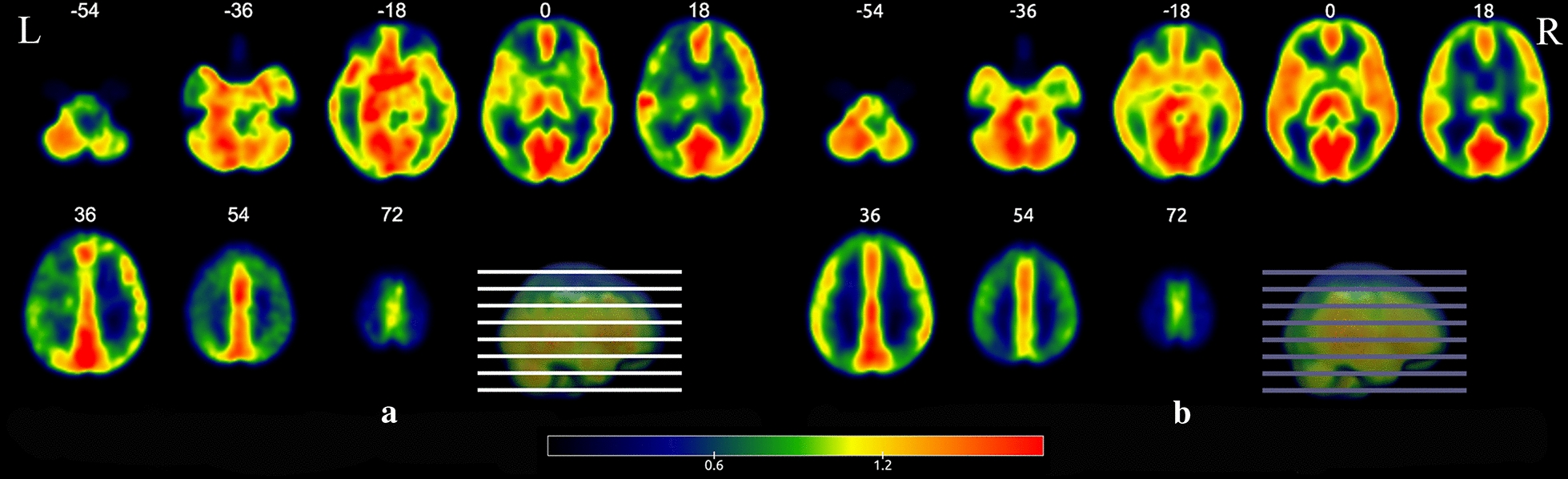
Average relative cerebral blood flow in patients with asymptomatic Moyamoya disease and healthy control subjects. **a** Patients with asymptomatic Moyamoya disease. **b** Healthy control subjects. -54, -36, 18 and so on represent the layers of the brain in the coronal plane

**Fig. 2:**
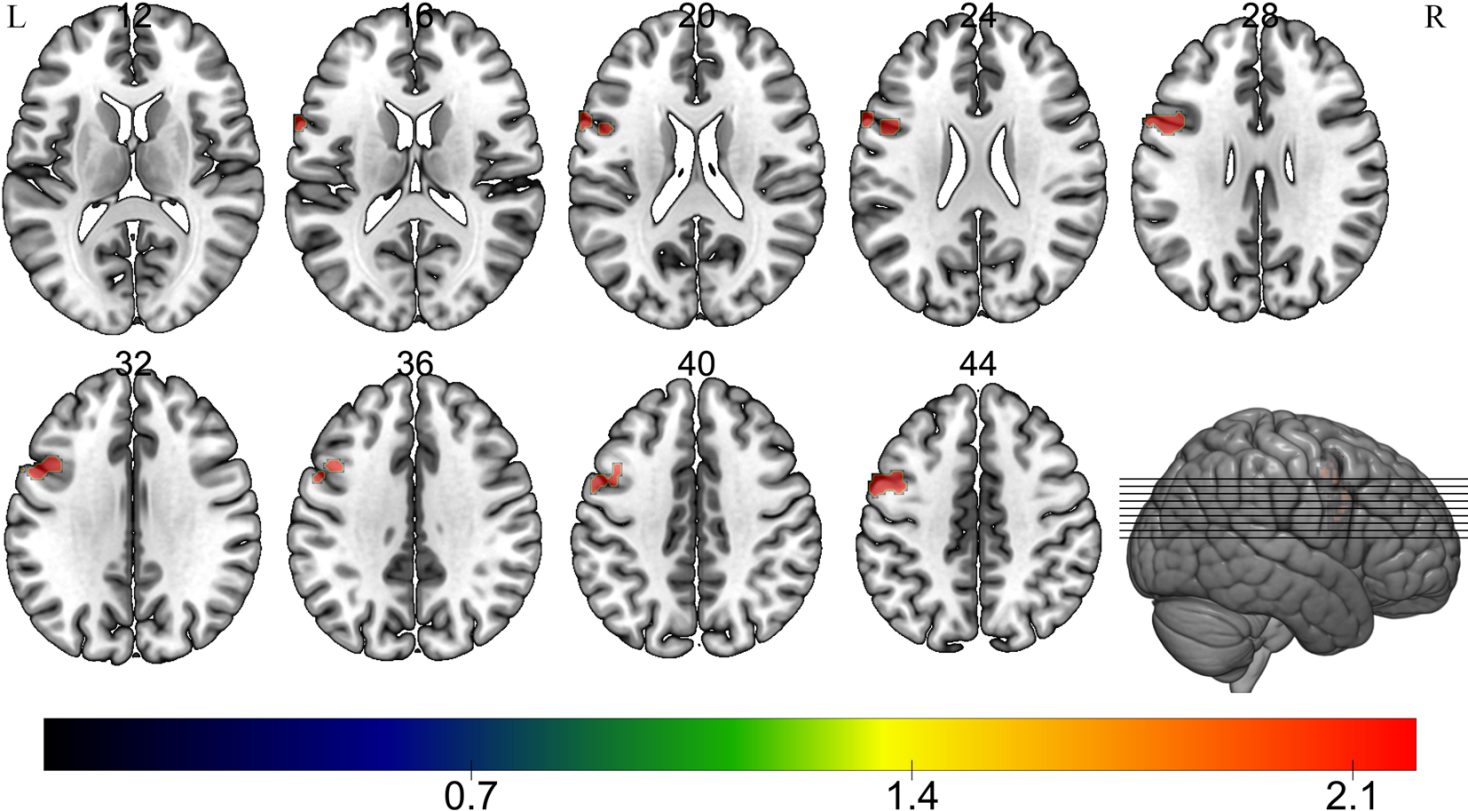
Difference in cerebral blood flow between the two study groups. The schematic diagram shows perfusion defects at various anatomical levels of the brain. 12, 16, 20 and so on represent the layer of the brain in the coronal plane. Multiple comparison of correction using family-wise error correction at the mass level (voxel level, *p* = 0.001)

The mean relative cerebral blood flow (CBF) is shown in Fig. 1. There was less CBF in the left anterior central and left inferior frontal gyri of the insula in the patients with asymptomatic MMD than in the control group (Fig. 2). The coordinates of the brain regions with abnormal CBF are shown in Table 2.

**Table 2:** Difference in cerebral blood flow between the two study groups and their location

MNI coordinate			Peak	Voxels, *n*	AAL region	Voxels in brain region, *n*
X	Y	Z				
− 44	14	34	4.3066	505	Precentral_L	297
					Frontal_Inf_Oper_L	144

*AAL* Anatomical Automatic Labeling brain atlas, *Frontal_Inf_Oper_L* left inferior frontal gyrus, opercular part, *MNI* Montreal Neurological Institute, *Precentral_L* left precentral gyrus

From Fig. 1b, which depicts mean cerebral blood perfusion in the control group, it can be deduced that there is an obvious perfusion defect in the left frontal lobe in the asymptomatic MMD group shown in Fig. 1a. The association between perfusion injury in the frontal lobe and cognitive impairment is discussed in the following sections.

### Differences in the executive control network

**Fig. 3:**
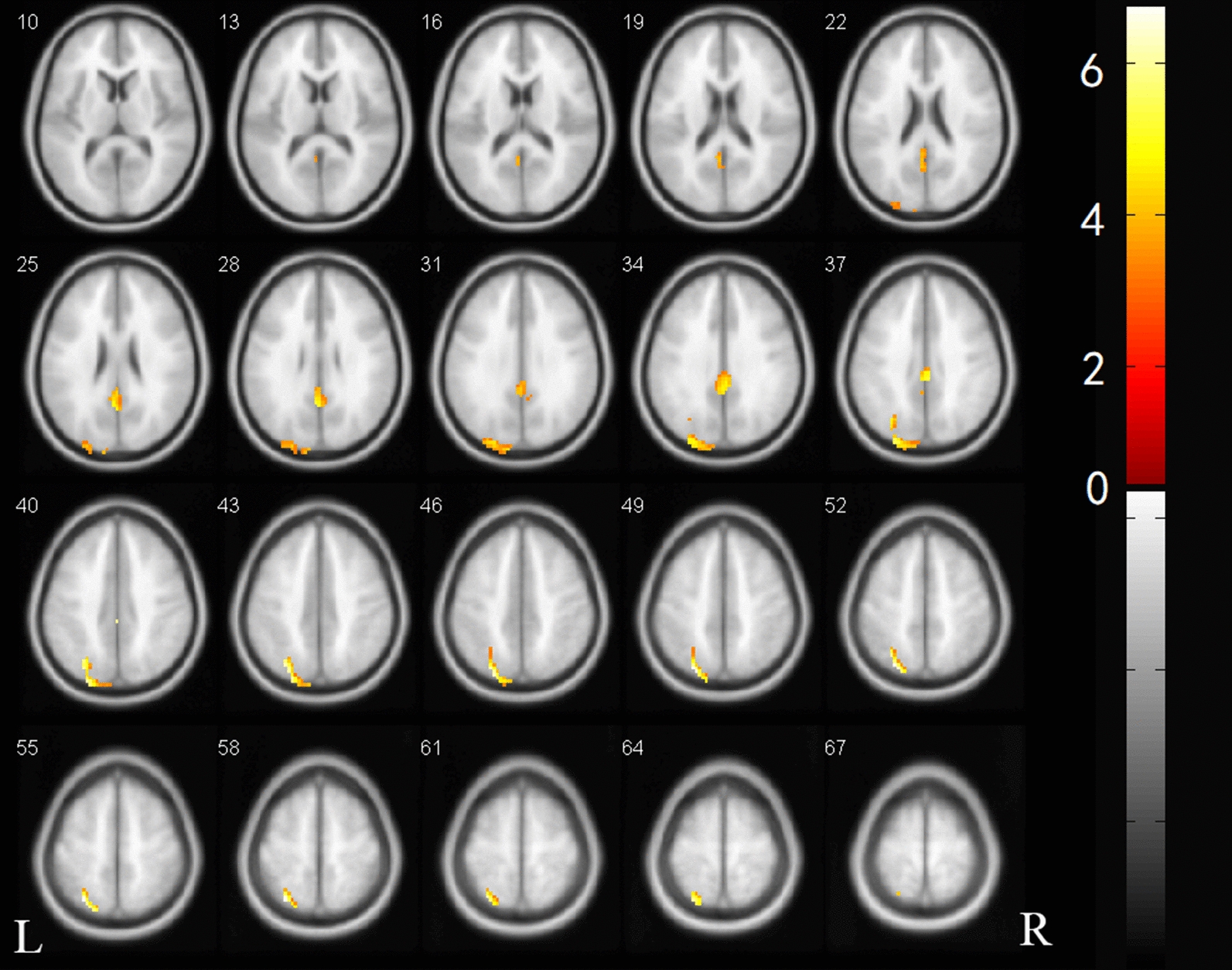
Difference in executive control network between the study groups. The two-sample *t*-test was used to detect differences in the corresponding network components between the two groups. Only the right side of each concerned network component performs the network component using family-wise error correction at a significance level of 0.05

In the independent component analysis, only the right executive control network survived after the correction; In this component, we found differences in the posterior cingulate gyrus, the left superior parietal gyrus, and the left superior occipital gyrus of the network (Fig. 3). The coordinates of the brain regions that were abnormal for executive control on the right are shown in Table 3.

**Table 3:** Differences in the executive control network between the two study groups and their location

MNI coordinate			Voxels, *n*	Peak	AAL region	Voxels in brain region, *n*
X	Y	Z				
0	− 33	39	113	6.1324	Cingulum_Post_L	34
− 30	− 66	54	254	7.2053	Parietal_Sup_L	82
					Occipital_Sup_L	64

*Cingulum_Post_L* left posterior cingulate gyrus, *Parietal_Sup_L *left superior parietal gyrus, *Occipital_Sup_L* left superior occipital gyrus

### Changes in node attributes

**Fig. 4:**
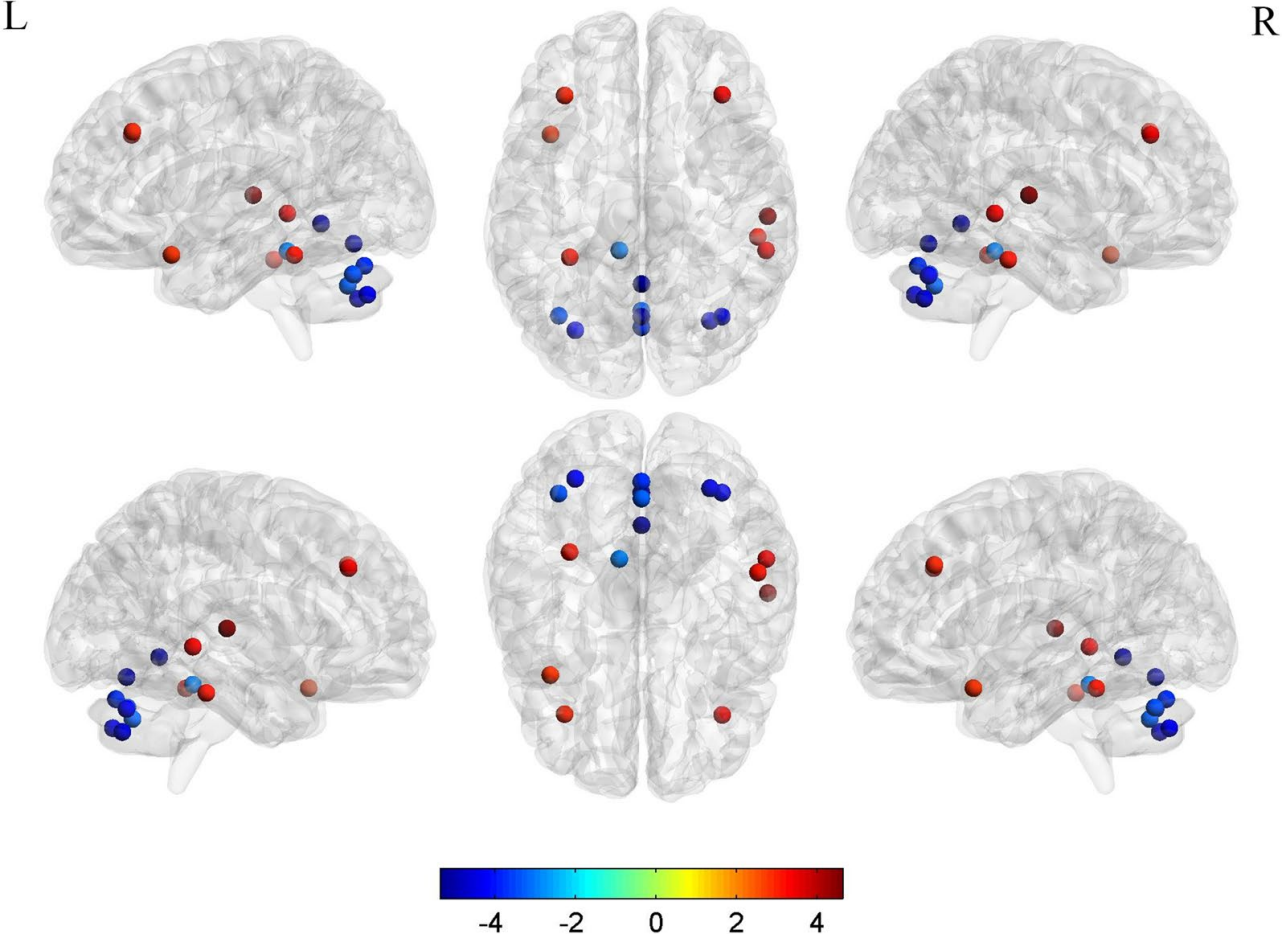
Difference in degree centrality between the two study groups. The value of the color bar represents the corresponding *t* value. The threshold of the images was at *p* < 0.05, corrected by the false discovery rate at a significance level of 0.01

There was a significant decrease in the degree centrality of the left and right medial frontal gyri, the fusiform gyrus and temporal pole of the left cerebral hemisphere, and the superior temporal gyrus, middle temporal gyrus, and inferior temporal gyrus of the right hemisphere (Fig. 4). Compared with the controls, patients with MMD had increased degree centrality in the cerebellum and vermis. The statistical charts and results for the other node attributes, including nodal efficiency and nodal local efficiency, are shown in Additional file 1: Figs. 1–6 and Tables 1–3.

### Changes in global network properties

Synchronization of global attributes obtained by graph theory analysis was significantly increased in patients with asymptomatic MMD in comparison with the control group (*p* = 0.037). There were no significant differences in the other four indicators. The mean value and standard deviation of specific indicators of global attributes are shown in Table 4.

**Table 4:** Differences in global attributes between the two study groups

	Control group (*n* = 20)	MMD group (*n* = 26)	*p* value
Assortativity	0.086 ± 0.044	0.088 ± 0.036	0.879
Hierarchy	0.032 ± 0.037	0.027 ± 0.029	0.491
Network efficiency	0.243 ± 0.012	0.249 ± 0.009	0.118
Synchronization	0.001 ± 0.005	0.005 ± 0.006	0.037
Small-worldness	0.632 ± 0.108	0.678 ± 0.084	0.126

Values are shown as the number (percentage) of cases unless otherwise indicated. The mean values are presented with the standard deviation

*MMD* Moyamoya disease, *SD* standard deviation

### Differences in brain functional connectivity between the study groups

**Fig. 5:**
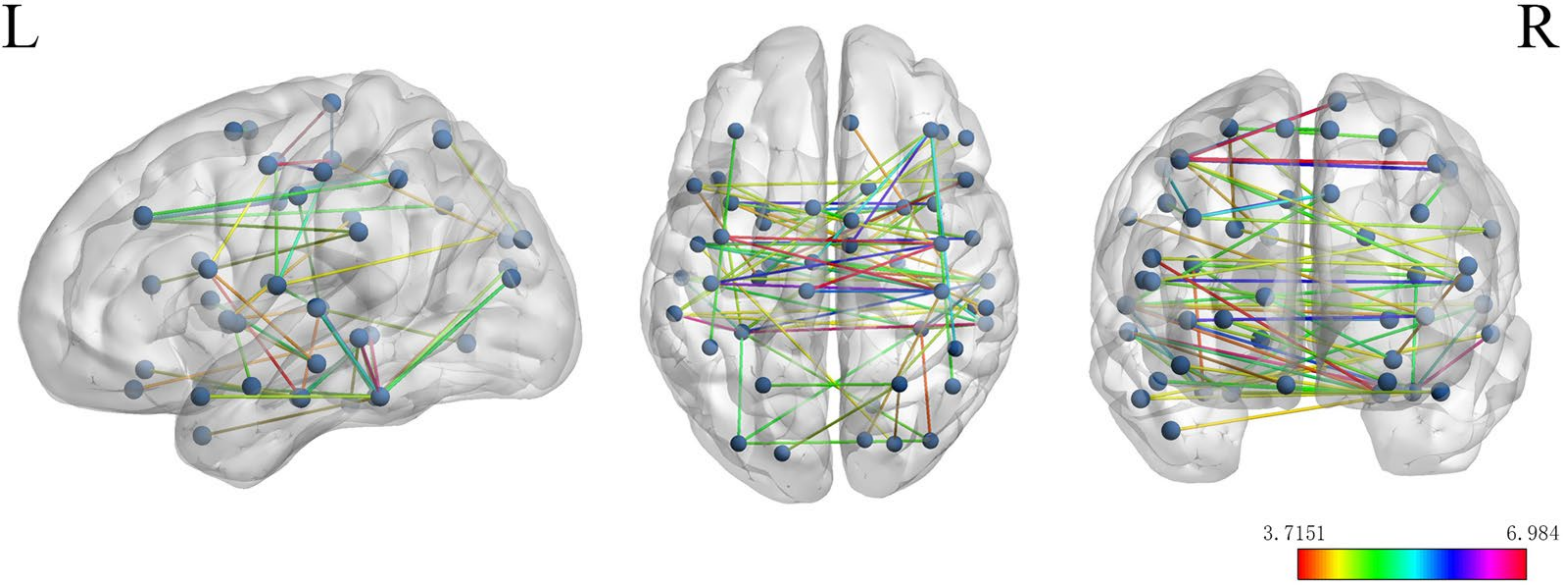
Differences in functional connectivity between the two study groups. The value of the color bar represents the corresponding *t* value. r threshold = 0.16, corrected by the false discovery rate at a significance level of 0.01

Figure 5 shows the difference in brain functional connectivity between the two study groups after correction of the false discovery rate. There were significantly fewer functional connections in the brain in the MMD group than in the control group.

### Relationship between imaging findings and neurocognition scores

The CBF was negatively correlated with complex subtraction (*r* = − 0.590, *p* = 0.044), and word short-term memory (*r* = − 0.643, *p* = 0.024) in patients with asymptomatic MMD. At the same time, in the patient group, synchronization of global attributes was negatively correlated with simple subtraction calculation (*r* = − 0.594, *p* = 0.042). However, other indicators such as nodes and edges had no significant correlation with cognitive scores.

## Discussion

This prospective integrated fMRI-based study is the first to elucidate the relationship between changes in brain perfusion, changes in the brain network, and cognitive impairment in patients with asymptomatic MMD. Our findings suggest that changes in CBF and networks in the brain are associated with cognitive impairment in patients with asymptomatic MMD.

### Abnormal cerebral perfusion and topology in patients with asymptomatic MMD

This study has demonstrated for the first time that synchronization is greater in patients with asymptomatic MMD than in normal controls. Synchronization measures how likely it is that all nodes fluctuate in the same wave pattern. This indicator is thought to play an important role in information processing in the brain [16].

The eigenratio of the graph Laplacian matrix is considered to be a common index of synchronization in complex networks [17]. Abnormalities in synchrony have previously been observed in EEG, MEG, and fMRI studies of schizophrenia [18, 19]. Jiang Y et al. found in their study that the synchronization of gray matter brain network was significantly negatively correlated with negative scores in The Positive and Negative Syndrome Scale. This part of the scale is often used to evaluate the stereotyped thinking, abstract thinking, etc. [20].

Furthermore, the index has a significant negative correlation with subtraction calculation (*p* = 0.042). The increase in synchrony in the asymptomatic moyamoya disease cohort may be associated with computational ability, but the association with complex subtraction does not meet statistically significant requirements and therefore requires further longitudinal analysis. Although neurosynchrony abnormalities may be a model of cognitive dysfunction for moyamoya disease like other models, they may reflect only one aspect of the disease.

Excluding synchronization, we found no significant differences in the remaining four indicators of global efficiency between the patients and controls. These findings are similar to those in patients with carotid artery stenosis, which is another type of ischemic disease, indicating that the cerebral blood supply has a strong compensatory influence in the chronic course of the disease [21]. Small-worldness (*S*) is thought to reconcile a large amount of short-range connections for segregation while maintaining a sufficient amount of long-range connections to assure integrated processing. The mean *S* score was slightly higher in the MMD group than in the control group (Table 4). This finding does not imply that the global efficiency was higher in the patients with MMD than in the controls but may reflect the vascular compensatory effect or some other mechanism in MMD.

There were significant between-group differences in nodal attributes, which may be considered sensitive indicators in this patient population. The node-based network parameters reflect the capacity of the network to transmit local information, energy consumption during transmission of information in the network, and the degree of network collectivization, all to different degrees. Our results indicate that the integrity and efficiency of functional networks may be jeopardized by varying degrees of damage and compensation after hemodynamic impairment. There was a significant decrease in degree centrality in the frontal and temporal lobes with a compensatory increase in the cerebellum in the MMD group but not in the control group. Degree centrality is the most direct measurement index for describing node centrality in network analysis. The larger the degree centrality, the more centrality the node has and the more important it is in the network. This phenomenon has not been identified in previous studies because most functional analyses have used an AAL90 template whereas in the present study we used an AAL116 template. The compensation of cerebellar nodes in patients with MMD may be related to compensated post-circulatory blood flow. More studies on cerebellar function are needed in these patients.

Using integrated analysis, we observed for the first time that patients with MMD have impaired frontal lobe perfusion, abnormal activation of the executive network, increased global synchronization of the brain, disrupted nodes and functional connectivity, and a number of abnormal indicators that are negatively correlated with cognitive scale scores. Patients with asymptomatic MMD have an increased risk of cognitive fragility and functional network impairment. Integrated fMRI analysis provides sensitive and objective information about brain perfusion and network connectivity related to cognitive impairment in these patients.

### Cognitive impairment in patients with asymptomatic MMD

First, there was a nonnegligible decline in intellectual reasoning ability in the patient group when compared with the control group, although the difference was not statistically significant (*p* = 0.06). Furthermore, there was a significant decrease in the patients' mathematical ability. The literature has shown that the ability to perform complex number operations is often associated with the parietal and frontal lobes [22].

In our patient group, CBF was significantly abnormal in the left inferior frontal gyrus and negatively correlated with complex subtraction test scores. A functional near-infrared spectroscopy study suggested that the frontal lobe is involved in complex mathematical calculations and that the left frontal lobe is activated by detailed processing while the right frontal lobe is activated by holistic processing [23]. This finding is consistent with that in other studies of the function of the frontal lobe and suggests that the change in CBF in the left frontal lobe may be an important reason for the decrease in the patients' computational ability.

Furthermore, the backward digit span task of verbal working memory is often associated with executive function, and a significant decline in executive function in patients with MMD has been reported [4, 24]. A similar decline was found in the present study.

We also found decreased activation in the posterior cingulate gyrus, the left superior parietal gyrus, and the left superior occipital gyrus in the right executive control network component (Fig. 3). This network includes multiple medial prefrontal cortex and inferior frontal and inferior parietal regions, with a core region known as the dorsolateral prefrontal cortex. The executive control network is associated with suppression of activity and mood. This network participates in several advanced cognitive tasks and plays an important role in adaptive cognitive control. Some studies have indicated that abnormal executive function may be related to a change in this network [25, 26]. Other studies have indicated that patients with MMD have abnormal executive function, but this study is the first to find impaired executive control network in patients with asymptomatic MMD.

In this study, there was no statistically significant difference in the short-term memory for image tests between the patients and the normal controls. We may find that some images are easier to remember than words, and education level may have less effect on short-term memory of images than that of words. Studies have also shown that decreases in memory of images become less pronounced over time [27, 28]. However, in our study, short-term memory for Chinese words decreased significantly and was negatively correlated with CBF in the patients with MMD. Four cognitive test results were correlated with the decline in CBF in the frontal lobe. The data from 15 patients in this study confirmed the hypothesis put forward in a previous study that included a small sample and showed impaired perfusion in the frontal lobes of patients with MMD and cognitive disorders [29]. It can be concluded that the frontal lobe controls a wide range of cognitive functions, and damage in this area has a considerable impact on neurocognition in patients with MMD [30]. More research is needed to delineate functional mapping of the frontal cortex.

## Limitation

This study has some limitations. Regarding ASL in moyamoya disease, elongated arterial transit artifacts in these patients made it difficult to quantitate true CBF using ASL [31, 32]. Also, rs-fMRI may not reflect brain function owing to the time delay in moyamoya disease [33, 34]. Therefore, we need to conduct related longitudinal studies with a larger sample of subjects to better clarify the differences in brain network and CBF between asymptomatic patients with moyamoya disease and the control group. Moreover, this study focused mainly on the changes in cerebral perfusion and brain function in patients with asymptomatic MMD, and further attention should be paid to the changes related to gray and white matter.

## Conclusions

Our findings show that these patients experience cognitive impairment in areas that include intelligence, executive function, short-term memory, and number manipulation, suggesting that cognitive impairment is a long-term complication in patients with asymptomatic MMD who receive conservative treatment. A left inferior frontal perfusion defect, increased cerebral synchronization, abnormal multi-node brain network, and decreased brain network connectivity may be important mechanisms of cognitive decline in asymptomatic MMD.

## Methods

### Participants

Participants in this prospective study included 26 asymptomatic patients with moyamoya disease who were admitted to our facility between January 2018 and December 2020. The asymptomatic patients included in this study were mainly those who came to the outpatient department of Beijing Tiantan Hospital for physical examination or with a family history of moyamoya disease. Fifteen patients were selected for more detailed cognitive tests after screening of MRI-T2 FLAIR images. After collection and analysis of the patients' baseline data, 20 healthy volunteers matched for age, sex, and years of education were recruited.

### Eligibility criteria

The inclusion criteria were as follows: (1) meeting of the criteria in the Japanese guidelines for diagnosis of MMD [35]; (2) for a patient with asymptomatic MMD, no previous ischemic or hemorrhagic attack, and in the case of intracranial lacunar cerebral infarction, a lesion diameter < 1.5 cm; (3) right-handedness. The following exclusion criteria were: (1) presence of another disorder that could affect cognitive function (e.g., Parkinson’s disease, Alzheimer’s disease); (2) a contraindication to MRI (e.g., a metal implant); (3) use of medication that could affect cognitive abilities; (4) fatigue, hunger, or inability to complete the tasks independently.

### Statistical analysis

For the cognitive analysis, the control group was matched with the patient group in terms of age, sex, education, and handedness. Analysis of variance was first performed to exclude an influence of sex on cognitive performance. A two-sample *t*-test was performed to examine differences between the study groups. All values are presented as the mean ± standard deviation. All participants' cognitive scores were correlated with significant brain network attributes by Pearson correlation analysis. All statistical analyses were performed using SPSS software, version 20.0 (IBM Corp, Armonk, NY). A *p*-value < 0.05 was considered statistically significant.

### Neuropsychological assessments and MRI data processing methods

For the neurocognitive function test, we adopted the method of He et al. [8]. Specific cognitive tests are detailed in the Additional file 1. The neuropsychologists, who had no knowledge of each patient's clinical data, used a computer workstation to test the study participants. Neuropsychological and fMRI examinations were separated by less than 5 days. Detailed MRI parameters and fMRI data processing procedures are shown in the Additional file 1.

The supplementary information includes an introduction to the cognitive function test scales covered in this study, as well as detailed parameters for the fMRI data, data preprocessing procedures, functional network analysis methods, and all references involved. A node diagram, a statistical table, and a histogram showing the differences in the three node attributes between the two groups are also included.

## Supplementary Information

**Additional file 1**: **Figure 1**. Difference in degree centrality between the two study groups. **Figure 2**. Histogram showing degree of centrality. G1, healthy controls; G2, patients with Moyamoya disease. **Figure 3**. Difference in nodal efficiency between the two study groups. **Figure 4**. Histogram showing nodal efficiency. G1, healthy controls; G2, patients with Moyamoya disease. The bar represents the mean and the errorbar represents the standard deviation. **Figure 5**. Difference in nodal local efficiency between the two study groups. **Figure 6**. Histogram showing nodal local efficiency. G1, healthy controls; G2, patients with Moyamoya disease. The bar represents the mean and the errorbar represents the standard deviation. **Table 1**. Differences in degree centrality between the study groups. **Table 2**. Differences in nodal efficiency between the study groups. **Table 3**. Difference in nodal local efficiency between the two study groups.

## Data Availability

All data generated or analyzed during this study are included in the published article. Some or all data, models, or code generated or used during the study are available from the corresponding author by request.

## Abbreviations

ASL: Arterial spin labeling
CBF: Cerebral blood flow
fMRI: Functional magnetic resonance imaging
HC: Healthy controls
MMD: Moyamoya disease
MRI: Magnetic resonance imaging
REST-IMP: ^123^I-N-isopropyl-p-iodoamphetamine
SD: Standard deviation

## References

[1] Shang S, Zhou D, Ya J, Li S, Yang Q, Ding Y (2020). Progress in Moyamoya disease. Neurosurg Rev.

[2] Suzuki J, Takaku A (1969). Cerebrovascular, “Moyamoya” disease: disease showing abnormal net-like vessels in base of brain. Arch Neurol.

[3] Kuroda S, Houkin K (2008). Moyamoya disease: current concepts and future perspectives. Lancet Neurol.

[4] Fang L, Huang J, Zhang Q, Chan RC, Wang R, Wan W (2016). Different aspects of dysexecutive syndrome in patients with Moyamoya disease and its clinical subtypes. J Neurol Surg.

[5] Kronenburg A, van den Berg E, van Schooneveld MM, Braun KPJ, Calviere L, van der Zwan A (2018). Cognitive functions in children and adults with Moyamoya vasculopathy: a systematic review and meta-analysis. J Stroke.

[6] Zhang P, Xu Q, Dai J, Wang J, Zhang N, Luo Y (2014). Dysfunction of affective network in post ischemic stroke depression: a resting-state functional magnetic resonance imaging study. BioMed Res Int.

[7] Pendlebury ST, Rothwell PM (2009). Prevalence, incidence, and factors associated with pre-stroke and post-stroke dementia: a systematic review and meta-analysis. Lancet Neurol.

[8] He S, Duan R, Liu Z, Ye X, Yuan L, Li T (2020). Characteristics of cognitive impairment in adult asymptomatic Moyamoya disease. BMC Neurol.

[9] Baba T, Houkin K, Kuroda S (2008). Novel epidemiological features of Moyamoya disease. J Neurol Neurosurg Psychiatry.

[10] Kuroda S; AMORE Study Group (2015). Asymptomatic Moyamoya disease: literature review and ongoing AMORE study. Neurol Med Chir (Tokyo).

[11] Stam CJ (2014). Modern network science of neurological disorders. Nat Rev Neurosci.

[12] Bassett DS, Zurn P, Gold JI (2018). On the nature and use of models in network neuroscience. Nat Rev Neurosci.

[13] Cheng W, Rolls ET, Gu H, Zhang J, Feng J (2015). Autism: reduced connectivity between cortical areas involved in face expression, theory of mind, and the sense of self. Brain.

[14] Eijlers AJ, Meijer KA, Wassenaar TM, Steenwijk MD, Uitdehaag BM, Barkhof F (2017). Increased default-mode network centrality in cognitively impaired multiple sclerosis patients. Neurology.

[15] Chang TY, Huang KL, Ho MY, Ho PS, Chang CH, Liu CH (2016). Graph theoretical analysis of functional networks and its relationship to cognitive decline in patients with carotid stenosis. J Cereb Blood Flow Metab.

[16] Fries P (2009). Neuronal gamma-band synchronization as a fundamental process in cortical computation. Annu Rev Neurosci.

[17] Barahona M, Pecora LM (2002). Synchronization in small-world systems. Phys Rev Lett.

[18] Ford JM, Krystal JH, Mathalon DH (2007). Neural synchrony in schizophrenia: from networks to new treatments. Schizophr Bull.

[19] Uhlhaas PJ, Haenschel C, Nikolic D, Singer W (2008). The role of oscillations and synchrony in cortical networks and their putative relevance for the pathophysiology of schizophrenia. Schizophr Bull.

[20] Jiang Y, Yao D, Zhou J, Tan Y, Huang H, Wang M (2020). Characteristics of disrupted topological organization in white matter functional connectome in schizophrenia. Psychol Med..

[21] Prabhakaran V, Rypma B, Gabrieli JDE (2001). Neural substrates of mathematical reasoning: a functional magnetic resonance imaging study of neocortical activation during performance of the necessary arithmetic operations test. Neuropsychology.

[22] Noguchi T, Kawashima M, Irie H, Ootsuka T, Nishihara M, Matsushima T (2011). Arterial spin-labeling MR imaging in Moyamoya disease compared with SPECT imaging. Eur J Radiol.

[23] Meiri H, Sela I, Nesher P, Izzetoglu M, Izzetoglu K, Onaral B (2012). Frontal lobe role in simple arithmetic calculations: an fNIR study. Neurosci Lett.

[24] Nakamizo A, Amano T, Michiwaki Y, Kawano Y, Kuwashiro T, Yasaka M (2018). Long-term neurocognitive outcomes in patients with adult Moyamoya disease. World Neurosurg.

[25] Oyegbile TO, VanMeter JW, Motamedi G, Zecavati N, Santos C, Chun CLE (2018). Executive dysfunction is associated with an altered executive control network in pediatric temporal lobe epilepsy. Epilepsy Behav.

[26] Dong G, Lin X, Potenza MN (2015). Decreased functional connectivity in an executive control network is related to impaired executive function in Internet gaming disorder. Prog Neuropsychopharmacol Biol Psychiatry.

[27] Morgan PL, Williams C, Ings FM, Hughes NC (2018). Effects of valent image-based secondary tasks on verbal working memory. Q J Exp Psychol (Hove).

[28] Goetschalckx L, Moors P, Wagemans J (2018). Image memorability across longer time intervals. Memory.

[29] Calviere L, Catalaa I, Marlats F, Viguier A, Bonneville F, Cognard C (2010). Correlation between cognitive impairment and cerebral hemodynamic disturbances on perfusion magnetic resonance imaging in European adults with Moyamoya disease. J Neurol Surg.

[30] Michalka SW, Kong L, Rosen ML, Shinn-Cunningham BG, Somers DC (2015). Short-term memory for space and time flexibly recruit complementary sensory-biased frontal lobe attention networks. Neuron.

[31] Fan AP, Guo J, Khalighi MM, Gulaka PK, Shen B, Park JH, Gandhi H, Holley D, Rutledge O, Singh P, Haywood T, Steinberg GK, Chin FT, Zaharchuk G (2017). Long-delay arterial spin labeling provides more accurate cerebral blood flow measurements in moyamoya patients: a simultaneous positron emission tomography/MRI study. Stroke.

[32] Hara S, Tanaka Y, Ueda Y, Hayashi S, Inaji M, Ishiwata K, Ishii K, Maehara T, Nariai T (2017). Noninvasive evaluation of CBF and perfusion delay of moyamoya disease using arterial spin-labeling MRI with multiple postlabeling delays: comparison with (15)O-Gas PET and DSC-MRI. AJNR Am J Neuroradiol.

[33] Kazumata K, Tha KK, Uchino H, Ito M, Nakayama N, Abumiya T (2017). Mapping altered brain connectivity and its clinical associations in adult moyamoya disease: a resting-state functional MRI study. PLoS ONE.

[34] Christen T, Jahanian H, Ni WW, Qiu D, Moseley ME, Zaharchuk G (2015). Noncontrast mapping of arterial delay and functional connectivity using resting-state functional MRI: a study in Moyamoya patients. J Magn Reson Imaging.

[35] Research Committee on the Pathology and Treatment of Spontaneous Occlusion of the Circle of Willis, Health Labour Sciences Research Grant for Research on Measures for Intractable Diseases. Guidelines for diagnosis and treatment of Moyamoya disease (spontaneous occlusion of the circle of Willis). Neurol Med Chir (Tokyo). 2012;52:245–66. 10.2176/nmc.52.245 22870528

