# Supplementary material for: Impairments in brain perfusion, executive control network, topological characteristics, and neurocognition in adult patients with asymptomatic Moyamoya disease
Source: BMC Neurosci. 2021 May 12;22:35. doi: 10.1186/s12868-021-00638-z (PMC8117595; doi:10.1186/s12868-021-00638-z)
Supplement: Supplementary file 1 — Additional file 1: Figure 1. Difference in degree centrality between the two study groups. Figure 2. Histogram showing degree of centrality. G1, healthy controls; G2, patients with Moyamoya disease. Figure 3. Difference in nodal efficiency between the two study groups. Figure 4. Histogram showing nodal efficiency. G1, healthy controls; G2, patients with Moyamoya disease. The bar represents the mean and the errorbar represents the standard deviation. Figure 5. Difference in nodal local efficiency between the two study groups. Figure 6. Histogram showing nodal local efficiency. G1, healthy controls; G2, patients with Moyamoya disease. The bar represents the mean and the errorbar represents the standard deviation. Table 1. Differences in degree centrality between the study groups. Table 2. Differences in nodal efficiency between the study groups. Table 3. Difference in nodal local efficiency between the two study groups. [file 12868_2021_638_MOESM1_ESM.pdf]

SUPPLEMENTARY MATERIALS

Supplementary information includes an introduction to the cognitive function test scales covered in this study, as well as detailed parameters of fMRI data, data preprocessing procedures, functional network analysis methods, and all references involved. In addition, the difference node diagram, statistical table and histogram of the three node attributes between the two groups are also included.

Acquisition of MRI scans

MRI data were obtained using a 3.0-Tesla MR system (Verio A Tim +Dot System, Siemens, Erlangen, Germany). A standard 12-channel head coil (3T Head MATRIX, A Tim Coil, Siemens) was used for signal reception. The subject lay supine with the head snugly secured by a belt and foam pads. For the resting state (rs)-fMRI scans, the subject was asked to close the eyes, not to fall asleep, and not to think about anything in particular. The scanning parameters were as follows: repetition time, 2220 ms; echo time, 30.0 ms; voxel size, 3.0 × 3.0 × 3.0 mm; field of view, 192 mm; slice thickness, 3.0 mm; number of slices, 32; and rs-fMRI scanning time, 11 min 27 s. A three-dimensional stack spiral fast spin echo sequence was used to obtain ASL (Arterial Spin Labeling) perfusion maps with the following parameters: bolus duration, 700 ms, h1990 ms; field of view, 192 mm; voxel size, 3.0 × 3.0 × 1.0 mm; slice thickness, 1.0 mm; repetition time, 5000 ms; echo time, 39.4 ms; and flip angle, 180°.

2.5 Data pre-processing

The rs-fMRI data were pre-processed using SPM 12 (Wellcome Department of Imaging Neuroscience, London, UK; https://www.fil.ion.ucl.ac.uk/spm/software/spm12/) implemented in MATLAB (Matlab Release 2013b, Mathworks Inc., Natick, MA, USA). The first six volumes of the individual functional images were discarded to achieve magnetization equilibrium. Slice-timing correction was implemented to align the rs-fMRI images according to the middle slice. The individual images were then realigned (standard of remove: 3 mm) so that each part of the brain was in the same position on all volumes and warped into the standard (Montreal Neurological Institute) MNI space by applying the transformation matrix derived by registering the T1 image (co-registered with functional images) into the MNI template using unified segmentation. Smoothing (4 × 4 × 4 mm) was used to improve the signal-to-noise ratio and to attenuate anatomical variances caused by inaccurate inter-subject registration after spatial normalization. Nuisance signals were removed from the time series of each voxel to reduce the effects of non-neuronal fluctuations, including head motion profiles and cerebrospinal fluid and white matter signals. The rs-fMRI data were bandpass-filtered to reduce the effects of low-frequency drift and high-frequency physiological noise. Regions of interest (ROIs) were placed using the Anatomical Automatic Labeling brain atlas, which divides the brain into 116 regions. Pearson's correlations for all time-course pairs were computed for each study participant and transformed into z-scores via Fisher's transformation.

2.6 Analysis of CBF

The analysis of cerebral blood flow (CBF) was reconstructed using the ASL_tbx.[1] Advanced normalization tools (https://sourceforge.net/projects/advants/) were used for one-step registration, CBF was registered on the PET template, and the voxel size was finally determined to be 2*2*2. ASL pre-processing was carried out by the data processing assistant (DPABI 4.3), and mean normalized segmentation was performed to obtain the relative CBF.[2] The CBF data were smoothed using a 6 × 6 × 6-mm smoothing box. The statistical method used was the two-sample *t*-test. The

covariables in the regression were sex, age, and scanning parameters. The whole brain mask was used to limit the statistical range and reduce the number of multiple comparisons. When a difference was found between the two study groups, multiple comparisons using FWE correction at the level of the voxel were performed. Finally, the MRIcroGL program (https://www.mccauslandcenter.sc.edu/mricrogl/) was used to draw the mean CBF images and compare them between the two study groups.

2.7 Functional network analysis

Graph theory analysis was performed using the following steps implemented in GRETNA software.[3] Pre-processed rs-fMRI images were structurally defined according to the Anatomical Automatic Labeling atlas. The mean time series was extracted from each parcellation unit, and pairwise functional connectivity values were estimated among the time series by calculating linear Pearson's correlation coefficients. After calculating the Pearson's correlation coefficient (r) for each ROIs, a 90 × 90 correlation matrix was constructed for each subject. For network analysis, various topological properties of the network were calculated using both global and nodal characteristics, which were compared with random network counterparts to determine non-randomness.

Six node-based and four global parameters were obtained for each network for graph theory analysis. The node-based network parameters included the nodal-clustering coefficient (C), shortest path length, nodal efficiency, nodal local efficiency (E loc), degree centrality (DC), and betweenness centrality (BC); the global parameters included network efficiency, small-worldness (s), assortativity (A), hierarchy, and synchronization. For edg, we take a time series between different brain regions and calculate the connections between them. The results were converted by Fisher Z, and then the GRETNA was used to perform a two-sample t-test between the edges for statistical analysis. Then visualize the resulting differences. Mathematical definitions of these parameters have been described elsewhere.[4]

The data were pre-processed by independent component analysis using the Group ICA in the fMRI toolbox (GIFT 4.0a, http://icatb.sourceforge.net/), which runs an Infomax algorithm. The pre-processed group data were decomposed into 47 spatially independent components. The data were concatenated and reduced using two-stage principal component analysis and the independent components, and then calculated using the Infomax algorithm. The GICA-3 back-reconstruction step was used to separate single-subject components from the set of aggregate components calculated in the previous step. Finally, for all subjects, the acquired spatial component maps were converted into z-score maps. The advanced visual processing network, left-sided and right-sided executive control network, front default mode network, and back default mode network were selected artificially according to a low-frequency to high-frequency energy ratio greater than 2 and the similarity with the spatial distribution of the component template; differences in the corresponding network components between the two groups of subjects were examined using the two-sample *t*-test.

Neuropsychological assessments

All cognitive assessments were performed using the Online Psychological Experimental System. Each task includes a practice session and a formal testing session. We followed the methods of He et al.(2020).[5]All tasks have shown acceptable half-split reliability values of 0.80–0.96 in previous studies.[6; 7; 8]

Nonverbal matrix reasoning was used to assess general intelligence and abstract reasoning ability, which has been correlated with mathematical performance.[9; 10] The task was adapted from Raven's

standard progressive matrices.[11] Each question has 4–6 potential responses and only one answer is correct. Participants were asked to identify the missing image from a sequence according to the rules behind it.

Mental rotation was used to evaluate visuospatial ability. The test was adapted from the study by Vandenberg and Kuse.[12] The revised version had only two choices and was limited to 3 min. Each trial had three three-dimensional images, one shown at the top of the screen and the other two at the bottom. Participants were asked to judge which of the two candidates at the bottom was the same as the top one after mentally rotating one of the images. The correct image was rotated from the original, with a rotation angle ranging from 15° to 345° (at 15°-intervals). The other image was a mirror image of the target. Participants pressed the "Q" key to select the image on the left side or "P" to select the image on the right side. An adjusted number of correct trials was used (see the sentence completion test).

A verbal working memory test was used to measure working memory capacity. The digit span test from the Wechsler intelligence scale was used. The test was divided into two parts, a forward digit span task representing short-term memory and general attention and a backward digit span task representing working memory related to executive function.[13; 14]

Simple subtraction was used to assess simple calculation ability and magnitude representation.[8] The task involved 92 subtraction problems with a correct single-digit answer. For each trial, a subtraction question (for example, 8-3) appeared at the top of the screen and two candidate answers at the bottom. The minuend of each question ranged from 2 to 18 and the answers ranged from 2 to 9. The official test time limit was 4 minutes.

The complex subtraction task had 95 problems, with each problem involving double-digit numbers for both operands. Most problems required mathematical borrowing. In each trial, a subtraction problem (e.g., 63–27) was presented at the top of the screen, with two candidate answers at the bottom. The difference between the true and false answers was 1 or 10. Formal testing of the task was limited to 3 min.

Word-memory ability and visual short-term memory were measured using the short-term memory span for Chinese words and phrases and the picture short-term memory test, respectively.[15] During the learning stage, a series of words and pictures was presented on the screen. In the test, the subjects judged whether the test images had been presented in the learning phase. If so, they pressed the "Q" key using their left index finger; otherwise, they pressed the "P" key with their right index finger.

The Edinburgh Handedness Inventory was used to investigate left and right handedness.[18] The subjects filled in the Edinburgh handedness questionnaire, where a final score ≥4 was classified as right handedness and scores ≤-4 were classified as left handedness; intermediate scores were classified as double handedness.[16]

The participants were tested by neuropsychologists, who were blinded to each patient's clinical data, using computer workstations. The interval between neuropsychological testing and MRI examination was <5 days. Conflict of Interest

The authors declare that the research was conducted in the absence of any commercial or financial relationships that could be construed as a potential conflict of interest.

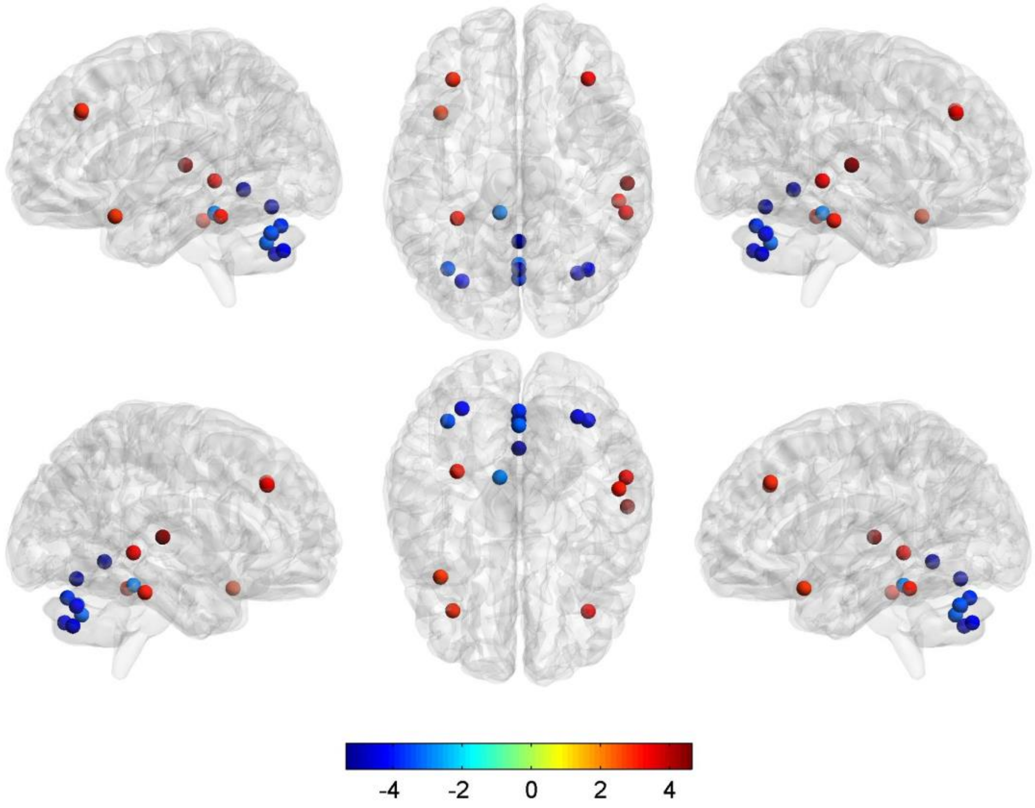

Supplementary Figure 1. Difference in degree centrality between the two study groups.

Supplementary Table 1. Differences in degree centrality between the study groups

	Control group (n=20)	MMD group (n=26)	p-value
MFG.L	19.24±4.04	15.09±4.41	0.0031
MFG.R	19.62±4.04	14.52±5.45	0.0009
FFG.L	22.26±3.41	17.53±5.58	0.0019
STG.R	24.08±4.20	17.15±6.04	0.0000
TPOsup.L	19.69±5.34	14.97±6.65	0.0054
MTG.R	24.59±4.69	19.01±6.09	0.0011
ITG.R	25.75±4.01	20.72±6.16	0.0030
CRBLCrus1.L	17.59±6.48	23.53±4.63	0.0009
CRBLCrus1.R	14.87±6.84	22.77±4.54	0.0000
CRBLCrus2.L	13.15±4.55	18.53±3.55	0.0001
CRBLCrus2.R	13.33±4.66	19.50±3.86	0.0000
CRBL3.L	1.76±1.79	4.70±4.34	0.0040

Vermis45	10.91±6.70	20.37±4.72	0.0000
Vermis6	8.69±6.78	18.16±5.44	0.0000
Vermis7	9.09±5.54	15.48±5.75	0.0004
Vermis8	7.65±4.75	12.47±4.95	0.0018

MMD, Moyamoya disease

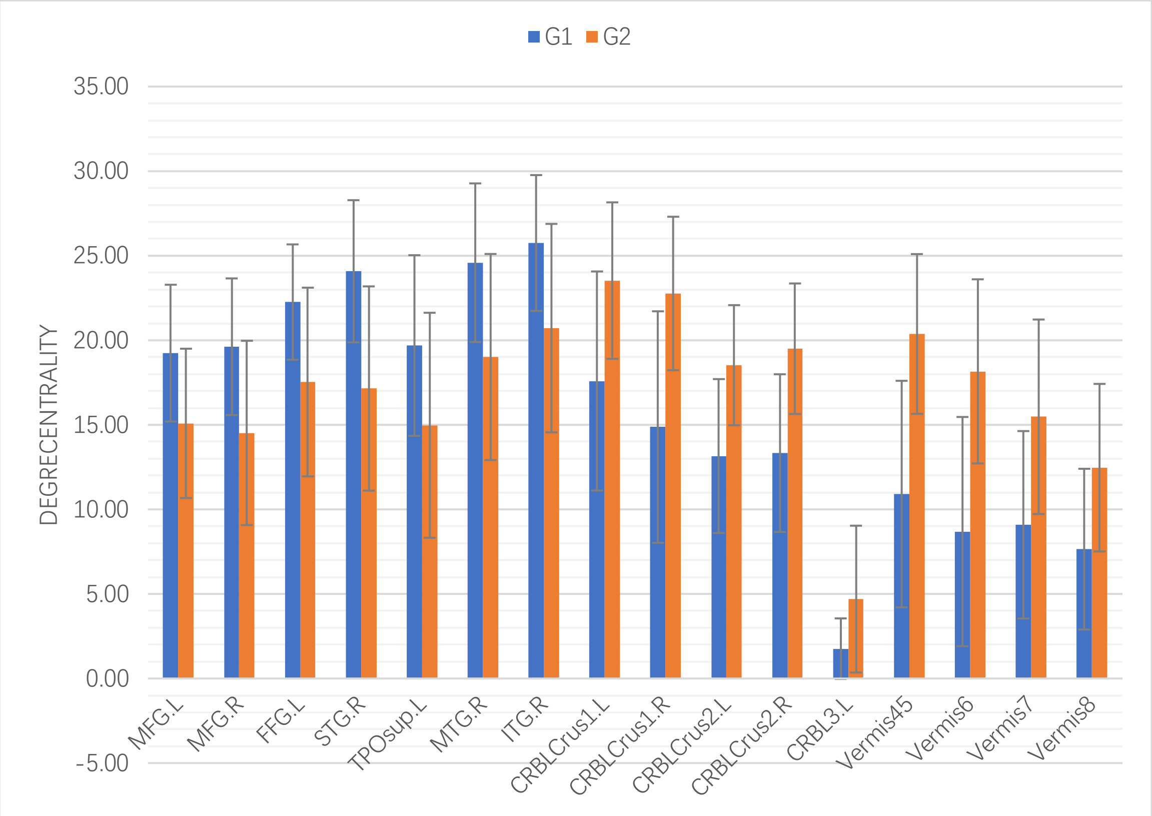

Supplementary Figure 2. Histogram showing degree of centrality. G1, healthy controls; G2, patients with Moyamoya disease

The bar represents the mean and the errorbar represents the standard deviation

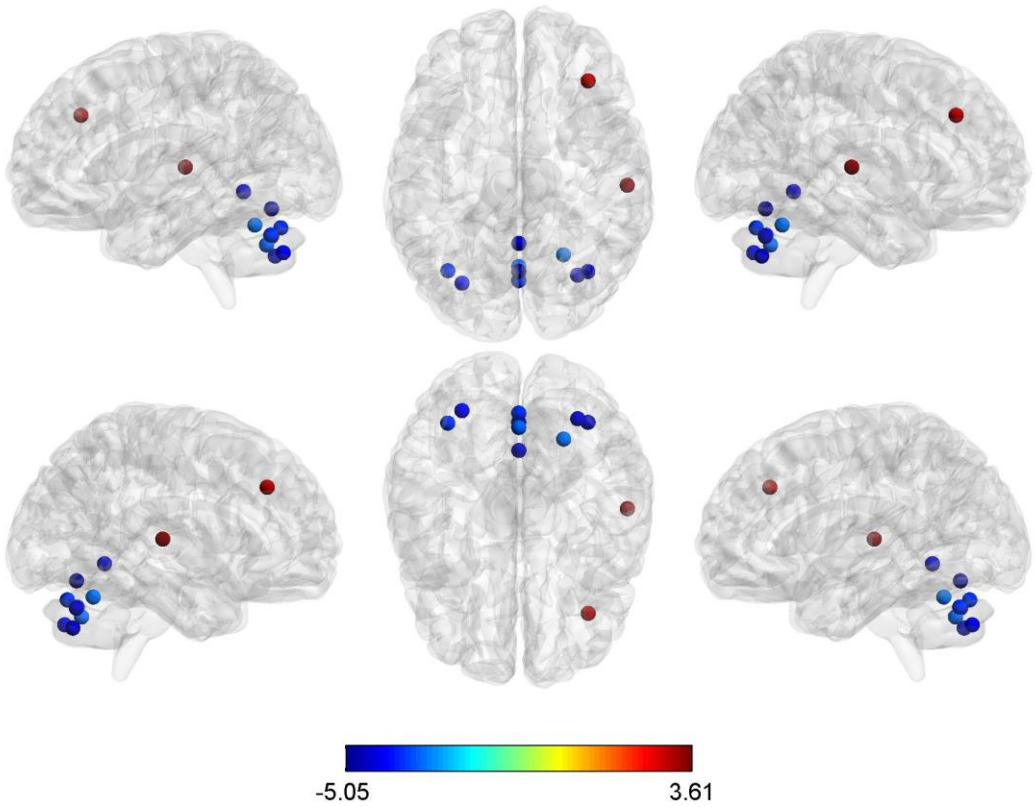

Supplementary Figure 3. Difference in nodal efficiency between the two study groups.

Supplementary Table 2. Differences in nodal efficiency between the study groups

	Control group (n=20)	MMD group (n=26)	p-value
MFG.R	0.28±0.02	0.26±0.03	0.0028
STG.R	0.31±0.02	0.27±0.04	0.0008
CRBLCrus1.L	0.27±0.04	0.31±0.02	0.0004
CRBLCrus1.R	0.26±0.04	0.30±0.02	0.0000
CRBLCrus2.L	0.25±0.03	0.28±0.02	0.0001
CRBLCrus2.R	0.25±0.03	0.29±0.02	0.0000
CRBL6.R	0.27±0.04	0.30±0.02	0.0020
Vermis4_5	0.22±0.06	0.29±0.03	0.0000
Vermis6	0.21±0.06	0.28±0.03	0.0000
Vermis7	0.21±0.06	0.26±0.04	0.0006
Vermis8	0.20±0.05	0.24±0.04	0.0016

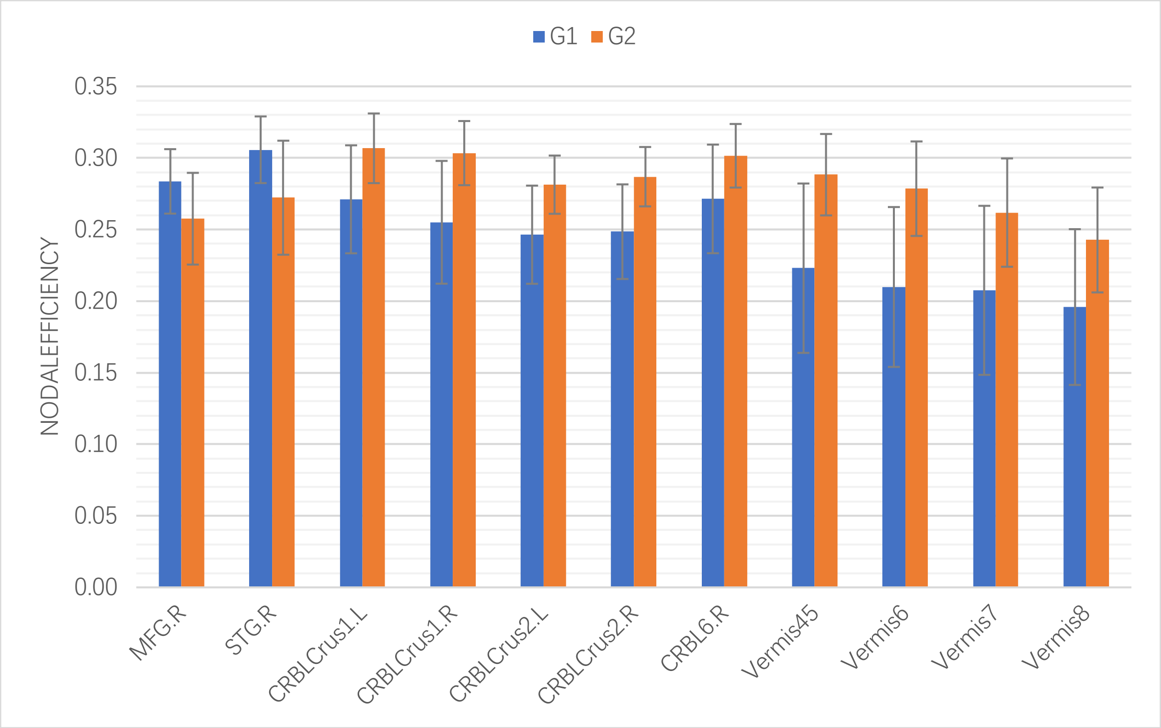

Supplementary Figure 4. Histogram showing nodal efficiency

G1, healthy controls; G2, patients with Moyamoya disease

The bar represents the mean and the errorbar represents the standard deviation

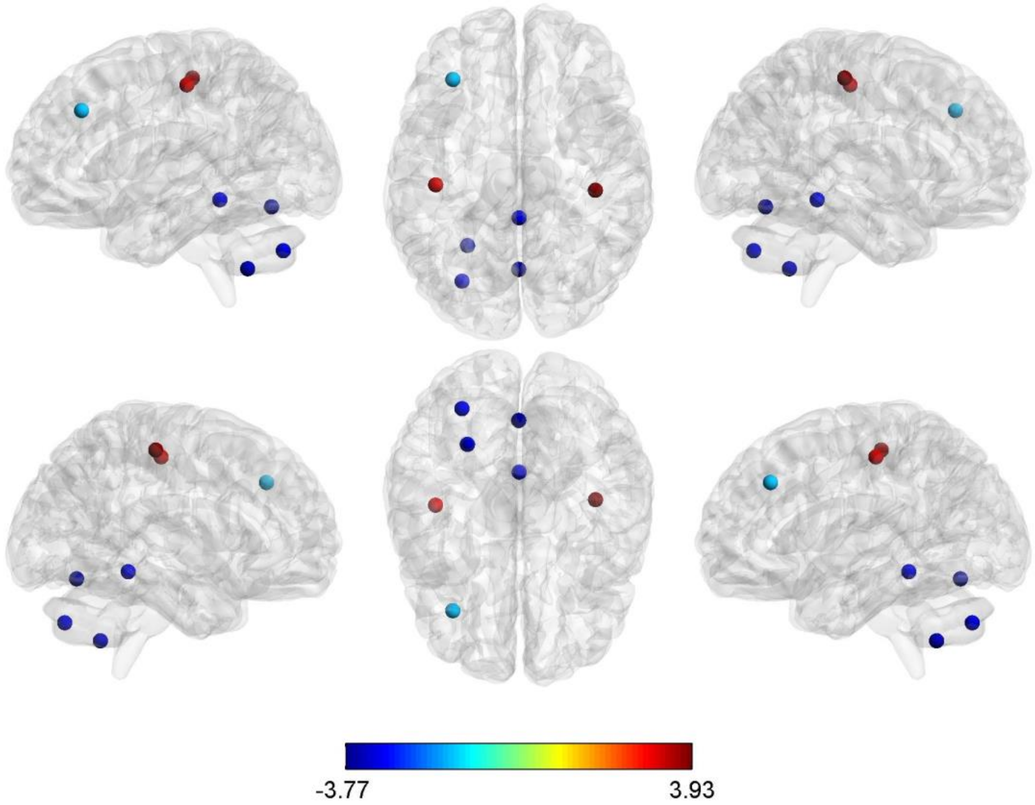

Supplementary Figure 5. Difference in nodal local efficiency between the two study groups.

Supplementary Table 3. Difference in nodal local efficiency between the two study groups.

	G1	G2	p-value
MFG.L	0.380546±0.01879	0.360301±0.023211	0.176996
PoCG.L	0.385339±0.017518	0.363624±0.025543	0.001189
PoCG.R	0.384444±0.017128	0.357709±0.02816	0.000306
CRBLCrus2.L	0.336375±0.041096	0.364949±0.019111	0.003054
CRBL8.L	0.321502±0.059198	0.365134±0.020529	0.001328
Vermis3	0.221285±0.118354	0.318475±0.086356	0.002864
Vermis6	0.30374±0.070012	0.362187±0.025332	0.000419

G1, healthy controls; G2, patients with Moyamoya disease

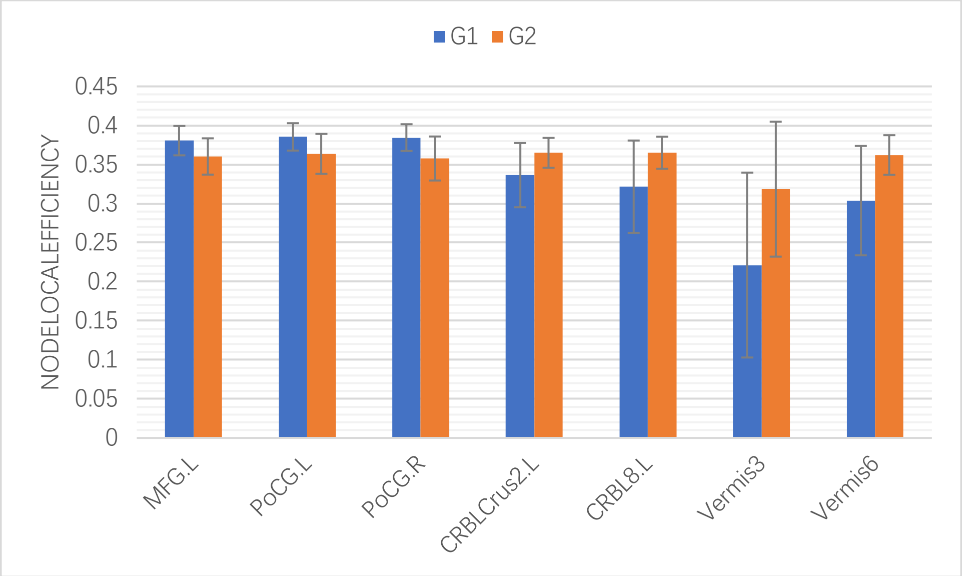

Supplementary Figure 6. Histogram showing nodal local efficiency

G1, healthy controls; G2, patients with Moyamoya disease

The bar represents the mean and the errorbar represents the standard deviation

References

[1] Z. Wang, G.K. Aguirre, H. Rao, J. Wang, M.A. Fernandez-Seara, A.R. Childress, and J.A. Detre, Empirical optimization of ASL data analysis using an ASL data processing toolbox: ASLtbx. Magn Reson Imaging 26 (2008) 261-9.

[2] C.G. Yan, X.D. Wang, X.N. Zuo, and Y.F. Zang, DPABI: Data Processing & Analysis for (Resting-State) Brain Imaging. Neuroinformatics 14 (2016) 339-51.

[3] J. Wang, X. Wang, M. Xia, X. Liao, A. Evans, and Y. He, GRETNA: a graph theoretical network analysis toolbox for imaging connectomics. Front Hum Neurosci 9 (2015) 386.

[4] M. Rubinov, and O. Sporns, Complex network measures of brain connectivity: uses and interpretations. Neuroimage 52 (2010) 1059-69.

[5] S. He, R. Duan, Z. Liu, X. Ye, L. Yuan, T. Li, C. Tan, J. Shao, S. Qin, and R. Wang, Characteristics of cognitive impairment in adult asymptomatic moyamoya disease. BMC Neurology 20 (2020).

[6] W. Wei, H. Lu, H. Zhao, C. Chen, Q. Dong, and X. Zhou, Gender Differences in Children’s Arithmetic Performance Are Accounted for by Gender Differences in Language Abilities. Psychological Science 23 (2012) 320-330.

[7] W. Wei, H. Yuan, C. Chen, and X. Zhou, Cognitive correlates of performance in advanced mathematics. Br J Educ Psychol 82 (2012) 157-81.

[8] X. Zhou, W. Wei, Y. Zhang, J. Cui, and C. Chen, Visual perception can account for the close relation between numerosity processing and computational fluency. Front Psychol 6 (2015) 1364.

[9] J.E.L. Minna Kyttälä, Some factors underlying mathematical performance: The role of visuospatial working memory and non-verbal intelligence. European Journal of Psychology of Education 23 (2008) 77-94.

[10] T.E. Rohde, and L.A. Thompson, Predicting academic achievement with cognitive ability. Intelligence 35 (2007) 83-92.

[11] J. Raven, The Raven's Progressive Matrices: Change and Stability over Culture and Time. Cognitive Psychology 41 (2000) 1-48.

[12] S.g.V.a.A.r. Kuse, Mental rotations, a group test of three-dimensional spatial visualization. Perceptual and Motor Skills 47 599-604.

[13] B.S. Heather M. Conklin, Clayton E. Curtis, Ph.D., Joanna Katsanis, Ph.D., and William G. Iacono, Ph.D., Verbal Working Memory Impairment in Schizophrenia Patients and Their First-Degree Relatives: Evidence From the Digit Span Task. Am J Psychiatry 157 (2000) 275–277.

[14] D.R. LaBelle, B.G. Lee, and J.B. Miller, Dissociation of Executive and Attentional Elements of the Digit Span Task in a Population of Older Adults: A Latent Class Analysis. Assessment 26 (2017) 1386-1398.

[15] J.Q. Yu Bolin, Stm span for chinese words and phrases. Acta Psychologica Sinica 17 (1985) 25-32.

[16] G. Dellatolas, M. De Agostini, F. Curt, H. Kremin, A. Letierce, J. Maccario, and J. Lellouch, Manual skill, hand skill asymmetry, and cognitive performances in young children. Laterality 8 (2003) 317-38.
